# Spectroscopic and Electrochemical Studies of Imogolite and Fe-Modified Imogolite Nanotubes

**DOI:** 10.3390/nano6020028

**Published:** 2016-02-02

**Authors:** Carmen Castro, Nicolas Arancibia-Miranda, Cristina Acuña-Rougier, Mauricio Escudey, Federico Tasca

**Affiliations:** 1 Department of Chemistry of Materials, University of Santiago of Chile, 9170022 Santiago, Chile; carmen.castroc@usach.cl (C.C.); nicolas.arancibia@usach.cl (N.A.-M.); cristiacu@gmail.com (C.A.-R.); mauricio.escudey@usach.cl (M.E.); 2 Center for the Development of Nanoscience and Nanotechnology, CEDENNA, 9170022 Santiago, Chile

**Keywords:** imogolite, Fe-modified imogolite, iron phthalocyanine, modified electrodes, oxygen reduction reaction

## Abstract

Carbon nanotubes and other forms of carbon nanoparticles, as well as metal nanoparticles have been widely used in film electrochemistry because they allow for the immobilization of larger amounts of catalyst (either biological or inorganic) on the top of the modified electrodes. Nevertheless, those nanoparticles present high costs of synthesis and of separation and purification that hamper their employment. On the other hand, imogolites (Im), with the general formula (OH)_3_Al_2_O_3_SiOH, are naturally-occurring nanomaterials, which can be obtained from glassy volcanic ash soils and can also be synthesized at mild conditions. In this research paper, we characterize through spectroscopic techniques (*i.e*., fourier transform infrared spectroscopy (FTIR) spectroscopy, powder X-ray diffraction (XRD) and transmission electron microscopy (TEM)) synthetized Im and Fe-modified imogolite (Im(Fe)). Moreover, the Im and Im(Fe) were physically adsorbed on the top of a graphite electrode (GE) and were characterized electrochemically in the potential region ranging from −0.8 to 0.8 V *vs.* the saturated calomel electrode (SCE). When the film of the Im or of the Im(Fe) was present on the top of the electrode, the intensity of the charging/discharging current increased two-fold, but no redox activity in the absence of O_2_ could be appreciated. To show that Im and Im(Fe) could be used as support for catalysts, iron phthalocyanine (FePc) was adsorbed on the top of the Im or Im(Fe) film, and the electrocatalytic activity towards the O_2_ reduction was measured. In the presence of the Im, the measured electrocatalytic current for O_2_ reduction increased 30%, and the overpotential drastically decreased by almost 100 mV, proving that the Im can act as a good support for the electrocatalysts.

## 1. Introduction

**Figure 1:**
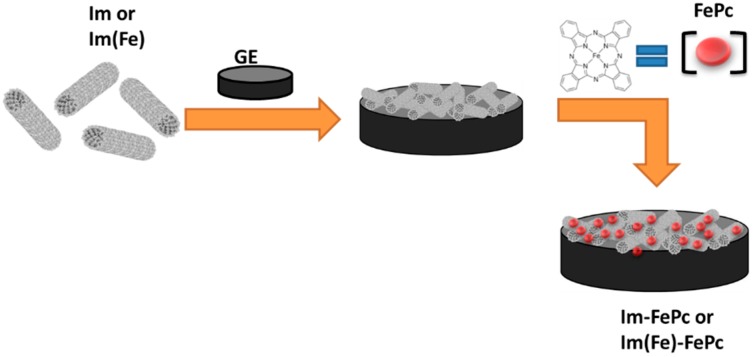
Scheme of the graphite electrode (GE) modified with imogolites (Im) or Im(Fe) and then with Im or Im(Fe) and FePc.

Nanoparticles are of great scientific interest because of their physical properties, like large surface-to-volume ratio, strong mechanical resistance and the presence of quantum effects. Because of all of those properties, they have been employed in various research fields, among them electrochemistry. Especially carbonaceous nanomaterials, like carbon nanotubes (CNTs) and graphene, have been extensively incorporated into various electrode architectures to increase the sensitivity of amperometric biosensors and sensors, to enhance the electron transfer from the redox center of proteins and inorganic molecules and to develop biological and inorganic anodes and cathodes [1,2,3,4]. The extremely high conductivity of those nanoparticles and their compatibility with both organic and inorganic molecules have made them excellent candidates for the development of electrochemical devices [2]. However, CNTs present the disadvantages of being hard to synthesize, separate, purify and modify, and they are expensive [5]. The high cost prohibits the massive use of CNTs for commercial products [1]. An interesting type of nanoparticle that could solve those problems is the imogolites (Im), which are naturally-occurring nanomaterials, with a structure similar to that of single-walled carbon nanotubes [6,7,8]. Im can be obtained from glassy volcanic ash soils and can also be synthesized [9,10,11]. They have the general formula (OH)_3_Al_2_O_3_SiOH, and tubes with various structures can be produced [8,12,13]. Moreover, different modifications can be applied to the inside and/or the outside of the tubes [10,14,15,16]. In nature, Im can be found as hollow nanotubes with an outer diameter of ~2.5 nm, an inner diameter of less than 1 nm and lengths between several hundred nanometers to one micrometer [11,17,18,19]. Im are regarded as a semiconductor when they are exposed to air [20]. However, it is known that Im react with phosphonates and other groups, like carboxylates [17], forming covalent bonds and giving products with different optical and electrical properties, which can be regarded as conductive [18,19,21,22,23,24]. In the present work, imogolite (Im) and Fe-modified imogolite (Im(Fe)) nanoparticles were synthesized and characterized by FTIR spectroscopy, powder X-ray diffraction (XRD), isoelectric point (IEP) and transmission and scanning electron microscopy (TEM, SEM). Moreover, the possibility of employing them as an electrochemical support was studied. Therefore, highly-oriented basal plane graphite electrodes (GE) modified with Im and Im(Fe) were characterized. The electrochemical window and potential limits of the modified electrodes immersed in phosphate buffer at pH 7 are studied by means of cyclic voltammetry in the presence of oxygen and in oxygen free buffer. To prove that Im and Im(Fe) can be employed as nano-supports for electrochemical devices, the Im and Im(Fe) nanoparticles were further modified with iron phthalocyanine (FePc) (see the schematic representation in Figure 1), and the efficiency of the electrochemical system for the oxygen reduction reaction (ORR) was studied [25]. To the best of our knowledge, this is the first time that Im and Im(Fe) have been employed as support nanomaterial for electrochemical devices.

## 2. Materials and Methods

All reagents and solvents were of analytical grade, obtained from Sigma-Aldrich (St. Louis, MO, USA) and used as received. For all of the experiments, deionized and double-distilled water was used. Im was prepared according to the procedure described by Arancibia-Miranda *et al.* [9,10]. Briefly, tetraethyl orthosilicate (TEOS) was added to a 5 mM aqueous solution of AlCl_3_ until an Al:Si ratio of 2:1 was reached. Then, a 0.1 M NaOH solution was added at a rate of 1.0 mL·min^−1^ until an Al:Si:OH ratio of 2:1:4 was obtained. The mixture was stirred during 60 min and then heated at 368 K for 5 days. Once the aging process was completed, the resultant mixture was allowed to cool down to ambient temperature. A 0.1 M NH_4_OH solution was added, stirring vigorously, until a pH of about 8.0 was reached. The solid was concentrated by centrifuging the suspension at 9000 rpm for 30 min and was washed with double distilled water until an electric conductivity lower than 0.78 S·m^−1^ was reached.

To prepare the Im(Fe), tetraethyl orthosilicate (TEOS) was added to a 150 mM aqueous solution containing a mixture of FeCl_3_ and AlCl_3_ in a molar ratio of Fe/(Al + Fe) = 0.05 [11]. The mixture was stirred for 1.5 h, and the gel-like precursor was obtained by slowly adding 0.1 M NaOH aqueous solution until a pH = 5.5 was reached. The salt-free precursor obtained after washing was dispersed in 2 L of double-distilled water. After adding 40 mL of 0.1 M HCl and stirring for several hours, the solution was kept at 373 K under sealing for 40 h. Both, the Im and Im(Fe) were dried at 373 K.

The isoelectric point (IEP) was determined through electrophoretic migration (EM) measurements, which were carried out with a Zetameter System 4.0 (Staunton, VA, USA). The imogolite samples (~100 mg) were suspended in 200 mL of 1.0 mM KNO_3_ aqueous solution; the EM was determined as a function of pH. From the plot of EM *versus* pH, the IEP was determined as the pH value at EM = 0.

Transmission electron microscopy (TEM): TEM samples were prepared by dipping Lacey-Carbon-Formvar-coated copper grids 300 Square, Pelco, (Redding, CA, USA) in dilute suspensions of Im or Im(Fe) and drying them in air before observation. Specimens were examined in an LEO 910 TEM (Zeiss, Oberkochen, Germany) operating at 120 kV.

X-ray diffraction (XRD): XRD analyses were carried out using oriented aggregate preparations obtained by drying water suspensions of the samples on glass slides. The samples were scanned from 3° to 70° 2θ using a step size of 0.02° 2θ and scanning for 1.0 s at each step. The X-ray patterns were collected using CuK α radiation from a Philips X’Pert diffractometer generator (Philips, Eindhoven, The Netherlands) and a theta/theta goniometer equipped with a 1.5° divergence slit, a 0.2° receiving slit, a graphite diffracted-beam monochromator and a scintillation counter [9,10].

SEM images of GE and GE modified with Im and Im(Fe) were recorded with a field emission scanning electron microscope (FE-SEM) Model 200 Nova FEI Company (Hillsboro, OR, USA). This equipment includes an energy dispersive X-ray detector Oxford INCA X-sight model (Oxfordshire, UK). The operative conditions were performed at low vacuum conditions, Helix detector at 10 to 18 kV. SEM images are shown in the Supporting Information file.

Fourier transform infrared spectroscopy (FTIR): FTIR spectra were obtained on a Tensor 27 Bruker spectrometer (Billerica, MA, USA) for both compounds by pressing 3 mg of dry sample in a spectral grade KBr matrix. The spectra were scanned 32 times at a resolution of 2 cm^−1^ [9,10].

Specific surface area (SSA): The SSA, and the micropore and mesopore volumes and diameter were determined from the nitrogen adsorption-desorption isotherms on a Micromeritics Model ASAP 2010 (Norcross, GA, USA) and in a Carlo Erba Sorptmatic 900 (Cornaredo, Italy) by the static volumetric method. Samples of 0.5 g were degassed at 483 K for 24 h, with a residual vacuum of 0.532 Pa. The SSA was calculated from the nitrogen adsorption isotherm at 77 K by the Brunauer, Emmett and Teller (BET) method through multipoint calculation by choosing the result given by the best linear fit in the 0.1 to 0.2 P/P° range [26,27]; the micropores’ volume was calculated from the nitrogen adsorption at 77 K by the t-plot method and derived by applying the α_s_ method; the volume and diameter of the mesopores were calculated from the adsorption/desorption nitrogen isotherm by the Barrett, Joyner and Halenda (BJH) method [26]. All samples were analyzed in triplicate.

For the electrochemical experiments, all of the solutions were purged with ultrapure nitrogen (N_2_) or ultrapure oxygen (O_2_) during 30 min prior to each measurement, depending on the experiment. Cyclic voltammetry was conducted between −0.8 and 0.8 V at 50 and at 5 mV/s. The working electrode was a highly-oriented basal plane pyrolytic graphite electrode (GE) (Pine Research Instrumentation, Durham, NC, USA) with a geometric area of 0.196 cm^2^ mounted on a Teflon support. The GE electrode was polished before each experiment with 1200 grit emery paper followed by ultrasonic treatment in purified water during 2 min [3].

The modification of the GE was performed by placing first 20 µL of 1 mg/mL nanoparticle suspension (*i.e*., Im or Im(Fe)) on the electrode and letting it dry to obtain a consistent and stable white-transparent film of nanoparticles, which was visible on the top of the GE (*i.e*., Im-GE or Im(Fe)-GE). To obtain electrodes modified with FePc, the previously-described Im-GE and Im(Fe)-GE were further modified by placing 20 µL of FePc solution until it dried. DMF was used to prepare 1 mM solutions of FePc.

A saturated calomel electrode (SCE) (Metrohm, Herisau, Switzerland) was used as the reference electrode, and the auxiliary electrode was a platinum (99.99%, Sigma-Aldrich) spiral wire with an exposed area of 10 cm^2^. The cyclic voltammetry experiments were performed with a Bio-Analytical Systems, BASI-Epsilon potentiostat (West Lafayette, IN, USA) using a conventional three-electrode electrochemical cell. A 0.1 M phosphate-buffer solution at pH 7 was employed during all of the experiments.

## 3. Discussion and Results

**Figure 2:**
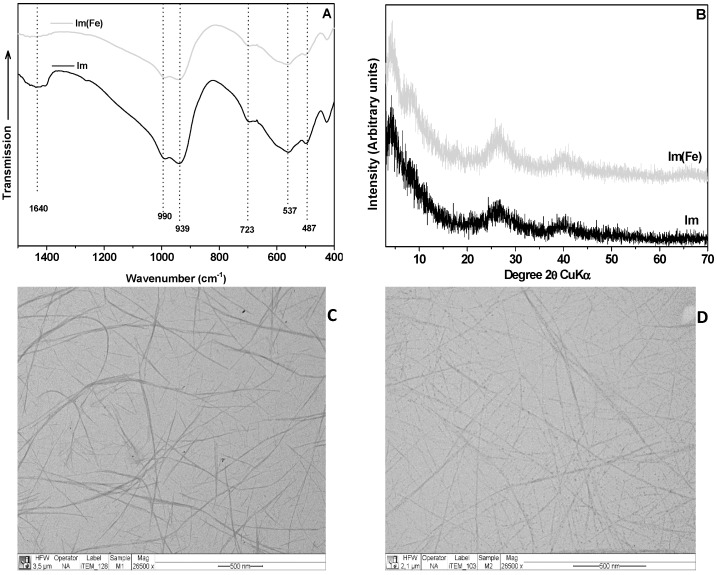
(**A**) Fourier transform infrared spectroscopy (FTIR) spectra of Im and Im(Fe); (**B**) X-ray diffraction (XRD) pattern of Im and Im(Fe); (**C**) Transmission electron microscopy (TEM) micrograph of Im; (**D**) TEM micrograph of Im(Fe).

**Figure 3:**
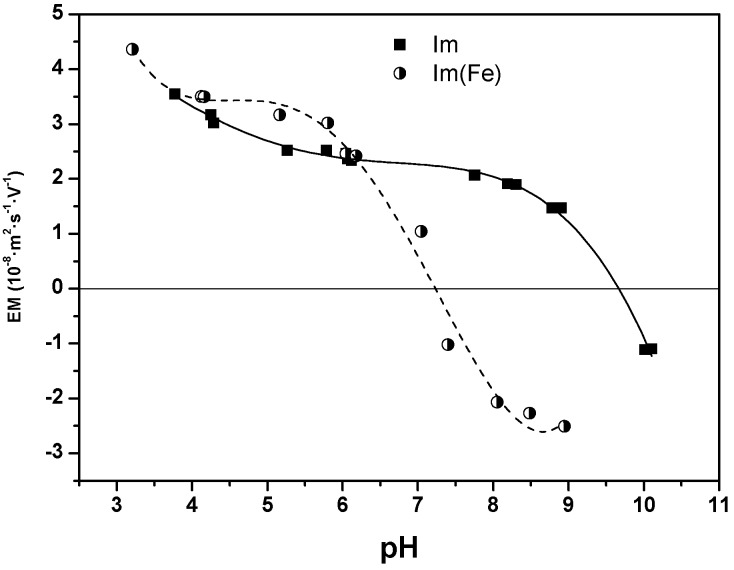
Electrophoretic migration *vs.* pH curves for Im (squares) and Im(Fe) (circles). EM, electrophoretic migration.

The surface of Im and Im(Fe) was characterized by FTIR, XRD, TEM and SEM. The features of the spectra indicate that both nanofibers have similar surface characteristics, with about 313 ± 15 and 303 ± 15 m^2^/g surface areas for Im and Im(Fe), respectively. Additionally, the pore volume (0.25 ± 0.01 cm^3^/g) and the micropore volume (0.02 ± 0.01 cm^3^/g) have the same values for both imogolites. The FTIR spectra of Im and Im(Fe) are shown in Figure 2A. Both samples show the characteristic signals of Im at 487, 537, 723, 990 and 939 cm^−1^, belonging to the Si-O stretching vibrations, which are specific for tubular structures. For the Im(Fe), the band width is greater compared to the Im, as a consequence of the larger size and weight of the Fe atom compared to the Al atom. The XRD patterns of Im and Im(Fe) exhibit four diffuse bands at 21.0, 12.0, 3.4 and 2.2 Å (Figure 2B). These reflections have been reported in the literature as characteristic for Im [9,28,29]. The IEP *vs.* pH curves are shown in Figure 3, with IEP_Im_ = 9.8 ± 0.2 and IEP_Im(Fe)_ = 7.3 ± 0.2. These results indicate that the synthesis of imogolite in the presence of Fe^3+^ results in a shifting of IEP values to more acidic pH values, resulting in differences in the magnitude of the surface charge from about pH = 6, and in the sign of the surface charge from pH over 7.3, consequently affecting the electrostatic interaction with charged species present in the solution.

The observation of the solid Im samples (Figure 2C) showed that the spatial distribution of the Im nanotubes resembles a spider web. The average dimensions of the Im exceed 500 nm in length. The micrograph of Im(Fe) is shown in Figure 2D.

Nanotube materials, 300 to 500 nm in length, are seen in these micrographs, and their fibrous morphology is similar to that of Im; nevertheless, the average length is half that of the Im. According to the analysis, the average diameter of Im is estimated at 2.0 to 2.1 nm, while the average diameter for Im(Fe) is estimated at 2.4 to 2.6 nm. The Im(Fe) presents also a small amount of spherical structure, which is probably due to the formation of allophane or Fe oxide. SEM images of bare GE (Figure S1) and GE modified with Im (Figure S2) and Im(Fe) (Figure S3) are shown in the Supporting Information file. Energy-dispersive X-ray spectroscopy analysis (EDX) shows the presence of Al and Si for the Im (Figure S2c), as well as some precursors employed for the synthesis (e.g., Na, K, Ca). For the Im(Fe), the presence of Fe is evident (Figure S3b,c).

**Figure 4:**
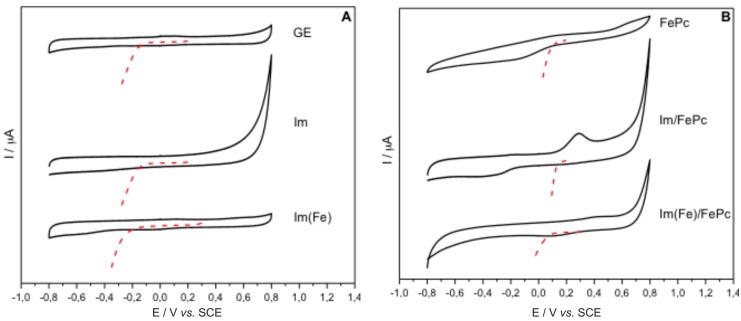
(**A**) Cyclic voltammetry and polarization curves for the oxygen reduction reaction (ORR) (O_2_ saturated conditions, dotted red line) of the graphite electrodes (GE) and GE modified with Im and with Im(Fe); (**B**) Cyclic voltammetry and polarization curves (O_2_ saturated conditions, dotted red line) of the GE modified with FePc, Im-FePc and Im(Fe)-FePc. SCE, saturated calomel electrode.

To prove that Im and Im(Fe) could be used as nanoparticles for electrochemical devices, GE were modified with 20 µL of 1 mg/mL water solutions of the respective nanoparticles. The modification of the GE was obtained by physical adsorption of the nanoparticles and is schematized in Figure 1. After the modification, a stable white-transparent film could be noticed on the top of the GE. The cyclic voltammetry in the absence and in the presence of oxygen for a bare GE and GE modified with Im and Im(Fe) is presented in Figure 4A.

Each cyclic voltammogram was repeated at least five times (with intervals of 30 m for depleting the O_2_ content with N_2_ or to saturate with O_2_) to guarantee the stability of the electrode, and at least five electrodes for each experiment were measured to guarantee the reproducibility of the experiments. Current density values varied from one electrode to another up to 10% of the total measured current. This difference was expected because of the various steps that are necessary for the preparation. In Figure 4A, the cyclic voltammograms for each kind of electrode modification (*i.e*., bare GE, GE modified with Im and GE modified with Im(Fe)) in the absence of O_2_ and in the presence of O_2_ (red curve) are presented. The results show that the electrodes modified with only Im and Im(Fe) do not present redox processes (Figure 4A) in the electrochemical potential window that ranges from –0.8 to 0.8 V *vs.* SCE. Extended X-ray Absorption Fine Structure studies (EXAFS) show that the Fe present in the Im(Fe) is octahedrally-coordinated, and a change in the oxidation state of the Fe^2+^ used as the precursor to Fe^3+^ occurs [30,31]. The magnitude of Fe signals in the EDX spectrum (Figure S3c) shows the low replacement of the Al atoms for Fe, which is consistent with the low amount allowed to maintain the nanotube structure [30]. The absence of redox processes in the presence of Im(Fe) is in accordance with the limited number of Fe atoms that can replace the Al present in the Im [11,30,31]. In the presence of imogolites, the electrode charging/discharging current (*i.e*., capacitive current) resembles the values that are double with respect to the unmodified GE. This is evidence of the increased active surface area. Similar results were obtained when GE were modified with CNTs [32,33,34]. Polarization curves in the presence of oxygen (Figure 4A) were recorded for the GE and for the modified GE. As we can notice from Figure 4A, in the presence of Im and Im(Fe), the overpotential for the ORR slightly decreases (≈50 mV towards more positive redox potentials with the ORR catalytic wave starting at −100 mV instead of −150 mV *vs.* SCE) in the presence of Im and Im(Fe). This effect can be explained by the difference of the exposed electro-active surface among the unmodified GE, the Im and Im(Fe)-modified GE. The high surface area of the Im and Im(Fe) could provide a more advantageous environment for the ORR. In fact, imogolites have been proposed as catalysts for various reactions before [11,35]. When GE are modified with only FePc, or with Im and FePc, or with Im(Fe) and FePc to form, respectively, Im/FePC and Im(Fe)/FePC, oxidation and reduction processes are noticeable at a potential around 200 mV for the FePC modified GE and at potentials of around 250 mV *vs.* SCE, when the imogolites are also present (Figure 4B and Figure S4). Those redox processes are attributable to the Fe^3+^/Fe^2+^ redox couple of the FePC. Furthermore, in the presence of the imogolites, the oxidation and reduction peaks have larger areas. While an increased area for the oxidation and reduction peaks was expected because of the higher active surface area due to the presence of the imogolites and, therefore, a higher concentration of the FePc on the top of the electrode, the displacement towards more oxidative potentials is a sign that more energy is required for the oxidation of the FePC, which is present on the top of the imogolites. Probably, the interactions of the imogolites with the FePC are rather repulsive, which could cause the increment of the formal redox potential of the FePC. Furthermore the presence of the imogolites could causes the formation of stacks which would require more energy to undergo the oxidation/reduction process and, therefore, the increment of the formal potential of the redox couple Fe^3+^/Fe^2+^ present in the FePc if compared to the formal potential of monolayers of FePc, which are present on the top of GE. Corresponding to an increased potential for the oxidation/reduction of the Fe^3+^/Fe^2+^ redox couple, also the potential for the ORR increased from ≈100 mV *vs.* SCE for the FePc GE system to ≈190 mV for the Im/FePc system and to ≈150 mV for the Im(Fe)/FePC. Indeed when imogolites are present, the overpotential for the ORR decreased by almost 100 mV. This is in agreement with the increased potential for the reduction of the Fe^3+^/Fe^2+^ redox couple. In fact, the latest research works of the ORR at phthalocyanine-modified electrodes suggest that closer the redox potential of the catalyst to the formal potential of the reaction to catalyze, the better the catalyst [36,37,38,39]. When the Im are combined with FePc, not only the overpotential for the ORR decreased, but also the catalytic current increased from 158 μA/cm^2^ for the FePc immobilized on GE to 226 μA/cm^2^ for Im/FePc and to 185 μA/cm^2^ for the Im(Fe)/FePc (in Figure S4, the entire catalytic curve is shown), proving again that the imogolites provide a large surface area, allowing a larger amount of active sites of FePc to concentrate on the top of the modified electrode surface. The Im proved to be a better support than the Im(Fe). Probably, the presence of Fe results in defects in the structure of the nanoparticles or the presence of Fe in the Im structure collides with the Fe of the FePc. Anyway, further experiments are necessary to explain the behavior of FePc on the top of Im-modified electrodes and also to understand the potential of imogolites in electrochemistry.

## 4. Conclusions

Im and Im(Fe) were characterized by FTIR, XRD, TEM and SEM and were evaluated by means of cyclic voltammetry and polarization curves for their employment in the development of electrochemical devices. Characterization of Im and Im(Fe) revealed that both nanoparticles have similar features: ≈300 m^2^/g surface area, pore volume of ≈0.25 cm^3^/g, average length of 500 nm and average diameter of ≈0.2 nm. Electrodes modified with Im and Im(Fe) showed an increased electrochemically-active surface area (increased capacitive current) and the absence of redox processes in the electrochemical window that ranges from −0.8 to 0.8 V *vs.* SCE. When GE were modified with Im or Im(Fe) and FePc, a redox peak for the oxidation-reduction of the Fe^3+^/Fe^2+^ redox couple was registered. The registered peak had a larger area than when FePc is absorbed on bare GE, proving that a large amount of FePc over the Im is absorbed and active. Furthermore, a displacement of the peak of ≈50 mV towards the oxidative region was noticed (from −150 to −100 mV *vs.* SCE). The modification with Im and FePc generated an increment of 30% in the current density for the ORR (≈220 μA/cm^2^), while with the Im(Fe)/FePc, only an increment of 15% was obtained (≈180 μA/cm^2^).

## Supplementary Materials

The following are available online at http://www.mdpi.com/2079-4991/6/2/28/s1. Figure S1: (a) Scanning electron microscopy (SEM) image and (b) energy-dispersive X-ray spectroscopy (EDX) spectrum of the graphite electrodes (GE) basal plane. Figure S2: (a) SEM image, (b) layered energy dispersed spectroscopy (EDS) and (c) EDX spectrum of GE modified with Im. Figure S3: (a) SEM image, (b) layered EDS and (c) EDX spectrum of GE modified with Im(Fe). Figure S4: (a) Cyclic voltammetry and polarization curves for the oxygen reduction reaction (ORR) (O_2_ saturated conditions, red line) of the GE and GE modified with Im and with Im(Fe); (b) cyclic voltammetry and polarization curves (O_2_ saturated conditions, red line) of the GE modified with FePc, Im/FePc and Im(Fe)/FePc.
