# Supplementary material for: Spectroscopic and Electrochemical Studies of Imogolite and Fe-Modified Imogolite Nanotubes
Source: Nanomaterials (Basel). 2016 Feb 2;6(2):28. doi: 10.3390/nano6020028 (PMC5302487; doi:10.3390/nano6020028)
Supplement: Supplementary file 1 [file nanomaterials-06-00028-s001.pdf]

Supplementary Materials: Spectroscopic and Electrochemical Studies of Imogolite and Fe-Modified Imogolite Nanotubes

Carmen Castro [1], Nicolas Arancibia-Miranda [1,2], Cristina Acuña-Rougier [1], Mauricio Escudey [1,2] and Federico Tasca [1,*]

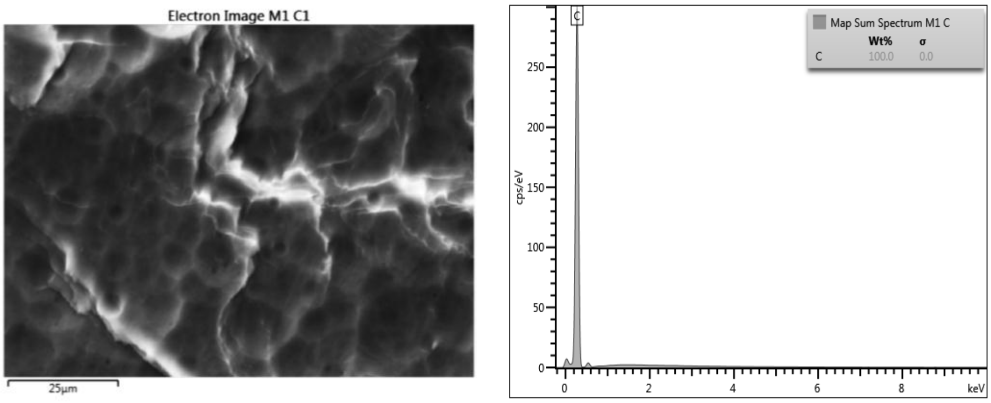

(**a**) (**b**)

Figure S1. (**a**) Scanning electron microscopy (SEM) image and (**b**) energy-dispersive X-ray spectroscopy (EDX) spectrum of the graphite electrodes (GE) basal plane.

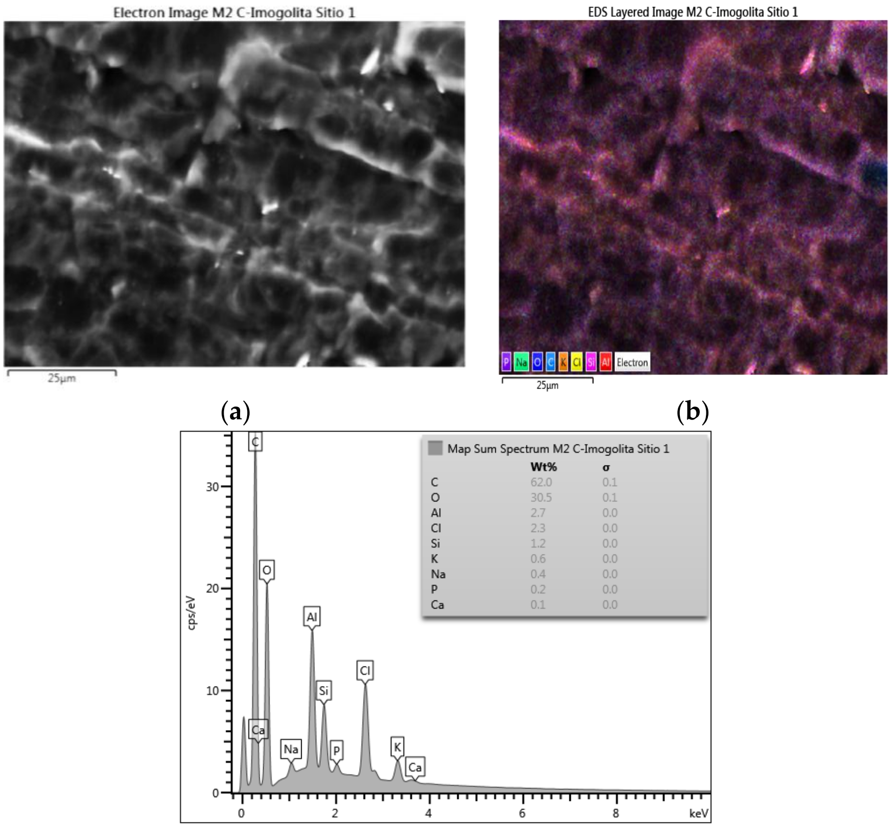

(**a**) (**b**)

(**c**)

Figure S2. (**a**) SEM image, (**b**) layered energy dispersed spectroscopy (EDS) and (**c**) EDX spectrum of GE modified with Im.

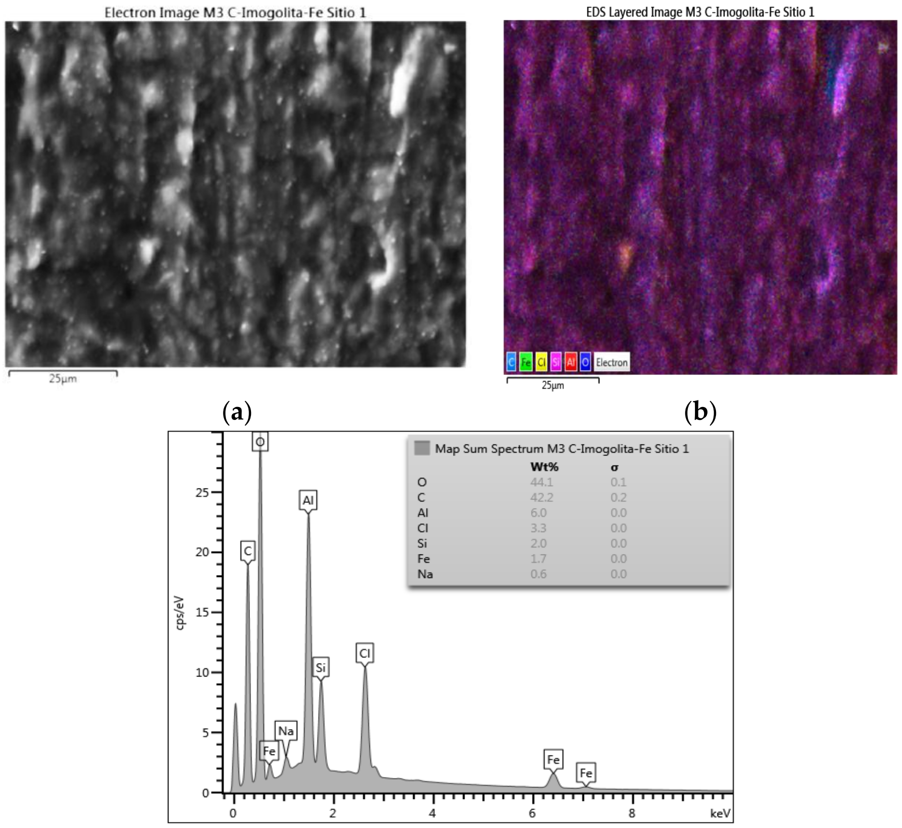

(**a**) (**b**)

(**c**)

Figure S3. (**a**) SEM image, (**b**) layered EDS and (**c**) EDX spectrum of GE modified with Im(Fe).

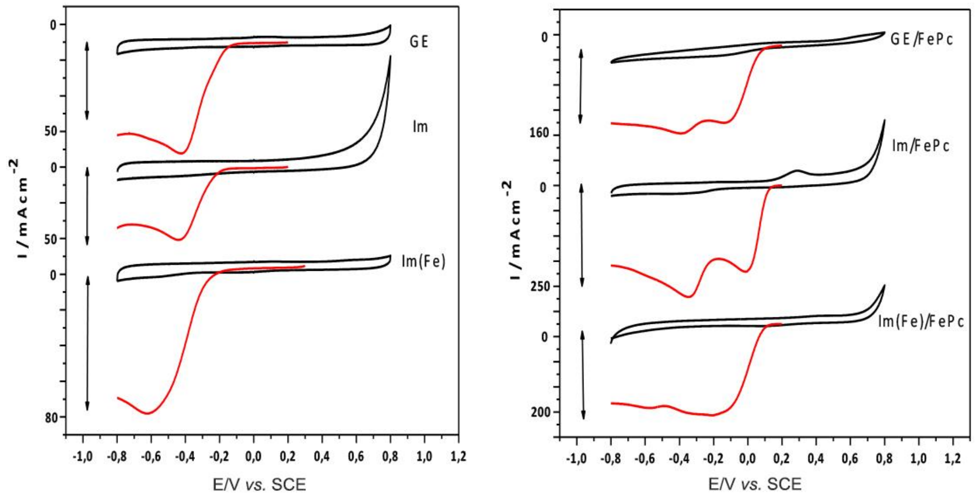

(**a**) (**b**)

Figure S4. (**a**) Cyclic voltammetry and polarization curves for the oxygen reduction reaction (ORR) (O_2 saturated conditions, red line) of the GE and GE modified with Im and with Im(Fe); (**b**) cyclic voltammetry and polarization curves (O_2 saturated conditions, red line) of the GE modified with FePc, Im/FePc and Im(Fe)/FePc.

© 2016 by the authors; licensee MDPI, Basel, Switzerland. This article is an open access article distributed under the terms and conditions of the Creative Commons by Attribution (CC-BY) license (http://creativecommons.org/licenses/by/4.0/).
